# Etymologia: Syphilis

**DOI:** 10.3201/eid1806.ET1806

**Published:** 2012-06

**Keywords:** etymologia, syphilis, French disease, Girolamo Fracastoro, Christopher Columbus, Europe, infection, Treponema pallidum, history

## Syphilis [′si-f(ə-)ləs]

From *Syphilis sive morbus gallicus* (“Syphilis or the French disease”) (1530) by Italian physician and poet Girolamo Fracastoro. The poem tells of Syphilus, a shepherd who insulted the sun god of Haiti. In retaliation, the god sends a plague to Haiti, and Syphilus is the first victim.

The first recorded syphilis epidemic was in 1495, during the First Italian War. After the French captured Naples, disbanded soldiers spread syphilis across Europe. For nearly 500 years, scholars have argued whether Columbus brought syphilis to Europe from the New World. Recent research supports Fracastoro’s New World origin for the disease.

## References

[1] Franzen C. Syphilis in composers and musicians—Mozart, Beethoven, Paganini, Shubert, Schumann, Smetana. Eur J Clin Microbiol Infect Dis. 2008;27:1151–7. 10.1007/s10096-008-0571-x 18592279

[2] Harper KN, Zuckerman MK, Harper ML, Kingston JD, Armelagos GJ. The origin and antiquity of syphilis revisited: an appraisal of Old World pre-Columbian evidence for treponemal infection. Am J Phys Anthropol. 2011;146(Suppl 53):99–133. 10.1002/ajpa.21613 22101689

[3] Quetel C. The history of syphilis. Baltimore: Johns Hopkins Press; 1990.

